# Glucocorticoids and antibiotics, how do they get together?

**DOI:** 10.15252/emmm.201505336

**Published:** 2015-06-15

**Authors:** Joachim Reidl, Eduard Monsó

**Affiliations:** 1 Institute of Molecular Biosciences, University of GrazGraz, Austria; 2 Servei de Pneumologia, Hospital Universitari Parc TaulíSabadell, Barcelona, Spain; 3 Ciber de Enfermedades Respiratorias – Ciberes, Universitat Autònoma de BarcelonaCerdanyola, Barcelona, Spain

## Abstract

Antibiotic therapy in patients currently treated with corticosteroids is common in chronic respiratory diseases when exacerbation symptoms attributable to infection appear. Among them, obstructive diseases such as asthma and chronic obstructive pulmonary disease (COPD) are major health issues affecting hundreds of million people worldwide that are frequently treated with inhaled corticosteroids. Systemic corticosteroids are also used for idiopathic pulmonary fibrosis, a less prevalent chronic respiratory disease. In this issue of *EMBO Molecular Medicine*, Earl *et al* (2015) report a potentially baleful relationship between steroid and antibiotic treatment in chronic respiratory diseases, affecting colonization persistence and antibiotic tolerance for *Haemophilus influenzae,* one of the leading potentially pathogenic microorganisms (PPMs) of the respiratory system.

See also: **CS Earl *et al*** (August 2015

Asthma and COPD are chronic inflammatory diseases that manifest as episodic or chronic dyspnoea, and have common characteristics in a proportion of patients, currently identified as asthma–COPD overlap syndrome (ACOS) (Bujarski *et al*, 2015). Both asthma and COPD patients with an ACOS clinical pattern show in most cases eosinophilic inflammation of their bronchial tree and respond well to corticosteroid treatment administered as a long-term inhaled therapy in most patients (Kew *et al*, 2014). Eosinophilic inflammation is in fact the most common host-response pattern in asthma (Haldar *et al*, 2008) and justifies the generalized use of corticosteroids to treat this disease. This treatment is also often used in COPD, is related to the appearance of pneumonia (Festic & Scanlon, 2015) and is currently restricted to patients with frequent exacerbations.

Bronchial colonization by PPMs is common in COPD (Rosell *et al*, 2005) as well as in severe asthma with partial reversibility and neutrophilic inflammation (Wenzel, 2012). In these diseases, chronic bacterial colonization is foremost composed of several bacterial species, including *Haemophilus influenzae*. Interaction between long-term corticosteroid treatment and bronchial colonizers has not been accurately assessed in patients, mainly due to the generalized use of this therapy in severe disease, making it difficult to compare with referent non-treated patient populations. As an emerging problem, patients with neutrophilic asthma poorly respond to steroid treatment and suffer from recurrent exacerbations, most of them due to bacterial infection (Biegelman *et al*, 2014). Under this scenario, elevated doses of steroids are often applied, which may cause severe side effects.

Antibiotic therapy failure correlates with bacterial biofilms. There is a high priority of awareness and many reports on bacterial biofilm-associated diseases exist describing their mechanisms on antibiotic tolerance. In the United States, an estimated 1.7 million hospital-acquired infections were reported annually and many of them are based on biofilm-related bacteria (Monina Klevens, 2007). The spectrum of biofilm-associated diseases is wide, and bacteria living in biofilm consortium show profound changes in lifestyle and metabolism that often preclude adequate targeting by antibiotic administration.

Treatment of COPD recurrent exacerbations, and of its ACOS phenotype, also combines steroids and antibiotics. This pharmacologic regimen is related to current therapy for asthma and includes bronchodilators, corticosteroids and antibiotics. In this clinical setting, the spectrum of PPMs associated with chronic colonization and exacerbations includes bacteria species such as *H. influenzae*, the microorganism addressed by Earl and colleagues who focused on the response of *H. influenzae* strains to the presence or absence of glucocorticosteroids.

Earl *et al* (2015) show that steroids promote an increased persistence of *H. influenzae* treated with beclomethasone, with enhanced bacterial load in the lungs of treated mice. To characterize the steroid response of bacteria, the impact of beclomethasone was determined at the transcriptome level of *H. influenzae*. Subsequently, bacterial genes were identified as significantly deregulated due to the presence of glucocorticosteroids. Among such genes were factors involved in virulence-associated functions such as iron uptake, biofilm formation, stress response, antimicrobial resistance and adherence. These bacterial genes were further identified as being expressed in glucocorticosteroid-treated lungs colonized by *H. influenzae*, indicating their responsiveness in the mouse colonization model. To prove whether steroid-responsive bacterial gene expression is also detectable in a clinical set-up, a cohort of patients was monitored and RNA tested for *H. influenzae* specific gene expression in sputum samples. The results show that all *H. influenzae*-colonized patients were positive for the corticosteroid-responsive bacterial genes.

To identify signal transduction components, which carry the glucocorticosteroid signal into the bacterial cell, a reporter strain was mutagenized. A panel of mutants was screened for loss of steroid response, yielding in a small subset of isolates mutated for genes involved in stress response and factors implicated in the adaptation of *H. influenzae* to lung infection. A most interesting gene candidate is a RpoE homolog, and RpoE-controlled genes are known to counteract extracytoplasmic stress in many bacteria. Ample knowledge exists for the RpoE regulon (Barchinger & Ades, 2013). For example, the system generates a response to physical, chemical or enzymatic caused stress conditions that target the outer membrane or periplasm. Earl *et al* (2015) characterized RpoE-dependent regulated genes in *H. influenzae* and show that glucocorticosteroids modify such response patterns, interfering with the RpoE network regulation. The study further shows that glucocorticosteroids impact biofilm formation and antibiotic tolerance: when glucocorticosteroid was added, biofilm development showed significant structural alterations (Fig1). Such modification correlated with increased tolerance to azithromycin, a commonly administered antibiotic in patients with asthma. Earl *et al* (2015) also tested a *rpoE* knockout mutant that indicated a similar trend to wild-type bacteria biofilm when exposed to glucocorticosteroids. Thus, evidence was provided whereby corticosteroids mediate phenotypes related to the RpoE signalling pathway. Finally, they convincingly show that *rpoE* knockout mutant behaves similarly for azithromycin tolerance, as compared with wild-type strain colonization in lung infection treated with glucocorticosteroids. Collectively, such data suggest that in the presence of glucocorticosteroids, the persistence of *H. influenzae* in the lung is increased and enhanced antibiotic tolerance is promoted (Fig1).

**Figure 1:**
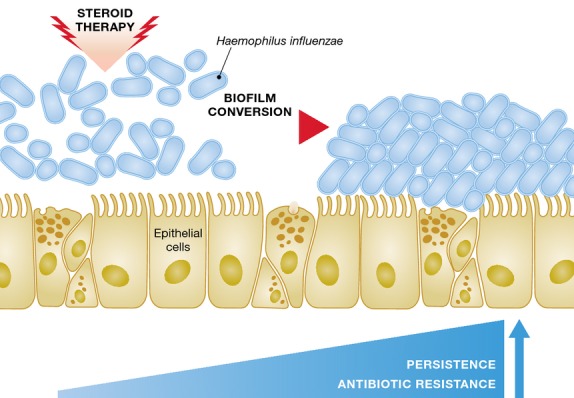
Steroid therapy and bacterial biofilm In the presence of steroids, biofilm conversion is taking place, which correlates with increased colonization persistence and antimicrobial resistance.

Abnormal local responses to the chronic presence of *H. influenzae* in the bronchial tree of chronic respiratory patients may be then partly mediated through inhaled corticosteroid treatment and, more importantly, current inhaled and systemic treatments may influence the pattern of changes in colonizing strains, determining, at least in part, the appearance of acute symptoms. The findings in the study of Earl *et al* (2015) may have significant clinical implications, considering the insights that recent research on bronchial microbiota has pointed out in chronic respiratory diseases, emphasizing the differential patterns of bronchial colonizers, related to severity and symptoms (Hilty *et al*, 2010; Millares *et al*, 2014).
